# Phytochemical inhibition of quorum sensing and biofilm formation by *Paederia foetida* Linn. against multidrug-resistant *Acinetobacter baumannii*: An integrated *in vitro* and *in silico* investigation

**DOI:** 10.14202/vetworld.2025.2181-2193

**Published:** 2025-08-02

**Authors:** Sirijan Santajit, Techit Thavorasak, Dararat Horpet, Thida Kong-ngoen, Uttapol Permpoon, Chul Young Kim, Tae-Gyu Nam, Nitaya Indrawattana

**Affiliations:** 1 Department of Medical Technology, School of Allied Health Sciences, Walailak University, Tha Sala, 80160, Thailand; 2 Center of Excellence in Tropical Pathobiology, Walailak University, Nakhon Si Thammarat, 80160, Thailand; 3 Department of Microbiology and Immunology, Faculty of Tropical Medicine, Mahidol University, Bangkok, 10400, Thailand; 4 Department of Pharmacy and Institute of Pharmaceutical Science and Technology, Hanyang University ERICA, Ansan, Gyeonggi-do, 15588, Republic of Korea; 5 Biomedical Research Incubator Unit, Department of Research, Faculty of Medicine Siriraj Hospital, Mahidol University, Bangkok, 10700, Thailand; 6 Siriraj Center of Research Excellence in Allergy and Immunology, Faculty of Medicine Siriraj Hospital, Mahidol University, Bangkok, 10700, Thailand; 7 Biodesign Innovation Program, Department of Parasitology, Faculty of Medicine Siriraj Hospital, Mahidol University, Bangkok, 10700, Thailand

**Keywords:** absorption, *Acinetobacter baumannii*, biofilm disruption, distribution, eugenol, excretion, gas chromato-graphy–mass spectrometry, metabolism, molecular docking, One Health, *Paederia foetida*, quorum sensing inhibition, toxicity

## Abstract

**Background and Aim:**

*Acinetobacter baumannii* is a multidrug-resistant (MDR) pathogen notorious for its biofilm formation and persistence in clinical and veterinary settings. Its resistance is exacerbated by quorum sensing (QS) pathways that regulate virulence and biofilm maturation. Disrupting QS and biofilm integrity using plant-derived compounds presents a promising alternative to traditional antibiotics. This study aimed to evaluate the antibiofilm and anti-QS potential of *Paederia foetida* Linn. ethanolic extract against *A. baumannii*, integrating gas chromatography–mass spectrometry (GC-MS) profiling, molecular docking, and *in vitro* assays.

**Materials and Methods:**

Leaves of *P. foetida* were extracted with ethanol and analyzed by GC-MS to identify major bioactive constituents. Molecular docking was conducted against five QS and biofilm-associated *A. baumannii* proteins (AF-A0A7S8WE28-F1-v4, AF-A0A059ZL64-F1-v4, AF-Q2VSW6-F1-v4, AF-A0A2P1B9S4-F1-v4, and AF-A0A5P9VY74-F1-v4). Absorption, distribution, metabolism, excretion, and toxicity (ADMET) profiles and drug-likeness of key compounds were assessed *in silico*. Antimicrobial activity was determined by broth microdilution (minimum inhibitory concentration [MIC]/minimum bactericidal concentration [MBC]), and biofilm inhibition was evaluated through crystal violet microtiter assays. Morphological damage was examined using field emission scanning electron microscopy (FE-SEM).

**Results:**

GC-MS identified 30 phytoconstituents, with 5-hydroxymethyl-2-furaldehyde, 4H-pyran-4-one derivative, and eugenol as predominant compounds. Eugenol exhibited the highest binding affinity, particularly with AbaR (−6.3 kcal/mol). The extract showed significant antimicrobial activity (MIC = 7.81 mg/mL; MBC = 31.25 mg/mL) and dose-dependent inhibition of biofilm biomass (p < 0.001). FE-SEM imaging confirmed dose-responsive membrane damage and disruption of the biofilm. ADMET predictions revealed favorable oral bioavailability and low toxicity for selected compounds.

**Conclusion:**

*P. foetida* extract exhibits potent antibacterial, anti-QS, and antibiofilm activity against MDR *A. baumannii*, supported by its phytochemical diversity, favorable pharmacokinetics, and strong protein-ligand interactions. These findings suggest its promise as a plant-derived therapeutic aligned with the One Health framework to combat antimicrobial resistance in both human and veterinary medicine.

## INTRODUCTION

*Acinetobacter baumannii* has become a growing concern in veterinary science due to its multidrug resistance, pronounced biofilm-forming capacity, and increasing prevalence in companion animals, livestock, and veterinary clinical environments [1, 2]. It is incre-asingly implicated in respiratory, wound, and systemic infections among animals [3]. A recent study involving 154 pets with respiratory symptoms identified a 6.5% prevalence of *A. baumannii*, with detection rates of 8.1% in dogs and 5% in cats, and 3.9% of these isolates were classified as multidrug-resistant (MDR). Additionally, environmental surveillance in veterinary hospitals revealed the presence of *Acinetobacter* spp. contamination on 6.0% of surfaces, including bedding and flooring materials used by dogs. The organism’s ability to persist in veterinary clinical environments and its genetic relatedness to human isolates highlight its zoonotic potential and capacity for cross-species transmission [4]. As the World Health Organization (WHO) priority pathogen, the presence of *A. bauma-nnii* in animal populations necessitates immediate intervention under the One Health framework, which emphasizes the interconnected health of humans, animals, and the environment. Effective strategies to control its spread in veterinary contexts must include improved surveillance, antimicrobial stewardship, and strict infection control protocols [5]. The ability of *A. baumannii* to form biofilms, regulated through quorum-sensing (QS) systems, significantly enhances its resistance to antibiotics, promotes persistence in host and environmental reservoirs, and contributes to its virulence. This makes QS inhibition (QSI) and biofilm disruption attractive targets for novel therapeutic interventions [6, 7].

Medicinal plants are increasingly anti-QS, and antibiofilm activities. *Paederia foetida* Linn., a member of the *Rubiaceae* family, has been used in traditional medicine for centuries to treat gastrointestinal (GI) disorders, infections, and inflammation [8, 9]. Recent research has revealed its rich phytochem-ical composition, including phenolics, flavonoids, and terpenoids–many of which exhibit QS inhibitory effects [5, 10]. However, the specific potential of *P. foetida* compounds in inhibiting QS pathways and disrupting biofilm formation in veterinary strains of *A. baumannii* remains largely unexplored. Phytoconstituents such as eugenol, thymol, salicylic acid, benzoic acid, and benzophenone–identified in crude extracts targeting poly-β-1,6-*N*-acetylglucosamine–have demonstrated the capacity to interfere with biofilm biosynthesis, weaken matrix integrity, and disrupt bacterial adhesion. Targeting key biofilm regulatory proteins such as AF-A0A2P1B9S4-F1-v4 (PgaA), AF-A0A5P9VY74-F1-v4 (PgaB), AF-Q2VSW6-F1-v4 (BfmR), QS regulators AF-A0A7S8WE28-F1-v4 (AbaI), and AF-A0A059ZL64-F1-v4 (AbaR) may increase *A. baumannii* susceptibility and present a viable cross-domain strategy for infection control [11–13].

Understanding the mechanisms by which plant-derived compounds interfere with QS and biofilm-associated pathways is essential for developing effective natural antibiofilm therapeutics. The crude ethanolic extract of *P. foetida* Linn. contains synergistic phytochemicals capable of modulating multiple molecular targets associated with biofilm formation and QS regulation. Additional constituents, including linoleic acid ethyl ester and phytol, are believed to disrupt bacterial communication and biofilm-associated signaling cascades, thereby destabilizing the microbial community structure [14, 15]. Despite growing recognition of *A. baumannii* as a critical pathogen within both human and veterinary contexts, current therapeutic strategies remain largely ineffective due to its robust biofilm formation and multidrug-resistant nature. While QS pathways play a central role in mediating biofilm development and antibiotic resistance, there is a lack of targeted interventions that can disrupt these regulatory networks without contributing to further resistance. Although medicinal plants such as *P. foetida* Linn. are known to contain bioactive compounds with antimicrobial potential, their specific roles in modulating QS systems and inhibiting biofilm-associated proteins in *A. baumannii*, especially in veterinary isolates, are underexplored. No integrated study has yet validated the anti-QS and antibiofilm efficacy of *P. foetida* using both *in vitro* assays and molecular docking approaches specific to veterinary strains of *A. baumannii*. This represents a significant gap in the development of sustainable, plant-based strategies for managing biofilm-associated infections in One Health settings.

This study aimed to investigate the anti-QS and antibiofilm potential of the ethanolic extract of *P. foetida* Linn. against multidrug-resistant *A. baumannii*. This was achieved by combining gas chromatography–mass spectrometry (GC-MS) for phytochemical profiling, *in silico* molecular docking of major compounds against key QS and biofilm-related proteins (AbaI, AbaR, BfmR, PgaA, and PgaB), and *in vitro* antimicrobial and biofilm inhibition assays. The findings of this study are intended to provide mechanistic insights and therapeutic evidence supporting the use of *P. foetida* as a natural, multi-target alternative to conventional antibiotics in veterinary and clinical settings under the One Health paradigm.

## MATERIALS AND METHODS

### Ethical approval

This study was approved by the Human Research Ethics Committee of Walailak University (WUEC-21-027-01) for the use of clinical bacterial isolates. No animal experimentation was performed.

### Study period and location

The study was conducted from March 2024 to February 2025 at Walailak University, Mahidol University (Thailand), and Hanyang University ERICA (South Korea).

### Bacterial strain isolation and identification

A clinical isolate of biofilm-forming *A. baumannii* was obtained from Maharaj Nakhon Si Thammarat Hospital, following ethical approval granted by the Human Research Ethics Committee of Walailak University (WUEC-21-027-01). The isolate was identified using standard microbiological procedures, including Gram staining, oxidase test, triple sugar iron test, Simmons citrate test, motility-indole-lysine test, and oxidative–fermentative tests. The confirmation of bacterial growth was performed on MacConkey agar incubated at 42°C [16].

### Plant collection and extract preparation

Fresh leaves of *P. foetida* Linn. were collected from Nakhon Si Thammarat District, Thailand (coordinates: 8.47102°N, 100.05395°E), and taxonomically identified by Dr. Prateep Panyadee from Queen Sirikit Botanic Garden, The Botanical Garden Organization, Chiang Mai, Thailand. A voucher specimen was deposited under QBG No. 133718. The leaves were washed thoroughly with distilled water, shade-dried at room temperature (25°C ± 2°C) for 7 days, and finely powdered using an electric blender (Philips, Netherlands). For extraction, 50 mg of the powdered material was dissolved in 1 mL of ethanol and subjected to ultrasonication for 30 min using an Elmasonic S30H unit (Elma, Germany) to enhance the release of phytochemicals. The extract was filtered using Whatman No. 1 filter paper (GE Healthcare, UK) and stored in amber vials at 4°C until further analysis [17].

### GC-MS analysis

Phytochemical constituents were analyzed using a GC-MS system (7890B GC–5977A MSD; Agilent Technologies, USA) equipped with a 70-eV electron ionization source. Separation was carried out on an HP-5ms capillary column (30 m × 250 μm × 0.25 μm), with helium (99.99%) as the carrier gas at a flow rate of 1.0 mL/min. The oven temperature program began at 50°C (held for 5 min), increased at a rate of 5°C/min to 300°C, and was then held at 300°C for an additional 5 min. A 1 μL aliquot of extract was injected in splitless mode. Mass spectra were recorded in the m/z range of 35–500. Compounds were identified by comparing their spectra with those in the Wiley23, NIST23, and NIST-2008 libraries (Agilent, USA), using a minimum match threshold of 80%. The relative abundance of compounds was determined based on the percentage of peak area.

### *In silico* absorption, distribution, metabolism, excretion, and toxicity (ADMET) profiling of anti-QS compounds

Major compounds identified by GC-MS were subjected to *in silico* ADMET analysis using the ADMETlab 2.0 webserver (https://admetmesh.scbdd.com, accessed on January 19, 2025). The analysis focused on parameters such as GI absorption, blood–brain barrier (BBB) permeability, cytochrome P450 (CYP450) enzyme inhibition, hepatotoxicity, and skin permeability [8, 18].

The selected major constituents–5-hydroxymethyl-2-furaldehyde (5-HMF), 2,3-dihydro-3,5-dihydroxy-6-methyl-4H-pyran-4-one (DDMP), and eugenol (all with ≥10% relative abundance)–were input into ADMETlab 2.0 using their SMILES notations. SwissADME (http://www.swissadme.ch, accessed on January 19, 2025) was used to evaluate drug-likeness and oral bioavailability based on standard filters including Lipinski’s Rule of Five, Veber, Egan, Ghose, and Muegge. Parameters such as water solubility, synthetic accessibility, and BBB penetration were also considered [19]. These evaluations informed compound prioritization for downstream bioactivity testing.

### Molecular docking simulation

The three-dimensional (3D) structures of five QS and biofilm-associated proteins from *A. baumannii*–AbaI, AbaR, BfmR, PgaA, and PgaB–were retrieved from the AlphaFold Protein Structure Database (https://alphafold.ebi.ac.uk; accessed January 21, 2025). The identifiers used were: AbaI, AbaR, BfmR, PgaA, and PgaB. Structures with predicted local distance difference test scores >80 were considered reliable. Structures were converted into pdbqt format using Open Babel version 3.3.1 (Open Babel Project, http://openbabel.org) [20] and prepared for docking through AutoDock 4.2 (The Scripps Research Institute, La Jolla, CA, USA) [21, 22].

Compounds with relative abundance ≥10% from the GC-MS profile and known anti-QS activity were selected as ligands. Their 3D structures were retrieved from the PubChem database (https://pubchem.ncbi.nlm.nih.gov; accessed on January 22, 2025). Docking simulations were performed using PyRx version 0.9.8 (Sargis Dallakyan and the Center for Computatio-nal Biology at The Scripps Research Institute) [23], which integrates AutoDock and AutoDock Vina with the Lamarckian Genetic Algorithm. Protein–ligand complexes with the lowest binding free energy (ΔG) were selected for analysis. Discovery Studio Visualizer 3.5 (BIOVIA, Dassault Systèmes, San Diego, CA, USA) was used to interpret molecular interactions such as hydrogen bonding, hydrophobic contacts, and electrostatic forces.

### Antimicrobial activity (minimum inhibitory concentration [MIC] and minimum bactericidal concentration [MBC] determination)

The MIC of *P. foetida* extract was determined using the broth microdilution method following CLSI guidelines [24]. Extract concentrations ranging from 62.50 to 0.12 mg/mL were prepared in a 96-well plate containing Mueller–Hinton Broth (MHB; Difco, Claix, France). Wells were inoculated with 100 μL of bacterial suspension (1 × 10^6^ colony forming unit/mL) and incubated at 37°C for 18 h. MIC values were determined using 0.03% resazurin dye (Thermo Fisher Scientific, UK); wells retaining blue color indicated inhibition of bacterial growth [25]. Tetracycline (30 μg/mL; Sigma-Aldrich, USA) and 1% dimethyl sulfoxide (DMSO; Merck, Germany) served as positive and negative controls, respectively.

For MBC determination, aliquots from wells without visible growth were streaked onto tryptic soy agar (TSA; Difco, USA) and incubated at 37°C. MBC was defined as the lowest extract concentration yielding no bacterial growth. All tests were performed in triplicate.

### Scanning electron microscopy (SEM) of treated bacteria

Morphological changes in *A. baumannii* cells exposed to *P. foetida* extract were analyzed by SEM, following minor modifications from published protocols [26]. Cultures were grown in Mueller–Hinton broth and treated with extract concentrations corresponding to MIC and MBC values. After 24 h incubation at 37°C, cells were pelleted by centrifugation (10,000× *g*, 5 min), placed on sterile glass coverslips (0.5 cm × 0.5 cm), and fixed in 2.5% glutaraldehyde (Sigma-Aldrich, USA) for 2 h. The samples were dehydrated in a graded ethanol series (20%–100%; RCI Labscan, Thailand), dried using a critical point dryer, and then coated with gold. Images were captured using a Zeiss EVO 10 SEM (Carl Zeiss, Munich, Germany) at the Center for Scientific and Technological Equipment, Walailak University. DMSO (1%) was used as the negative control.

### Biofilm inhibition assay

The anti-biofilm activity of *P. foetida* extract was evaluated using a crystal violet (CV) microtiter plate assay [27]. *A. baumannii* was grown in Luria–Bertani (LB) broth (Himedia, India) at 37°C for 24 h. The culture was diluted 1:100 in fresh LB medium containing 1% glucose, and 200 μL of this suspension (adjusted to 0.5 McFarland standard) was added to 96-well plates (Nunc, Thermo Fisher Scientific, USA). Plates were incubated at 37°C for 24 h to allow biofilm formation.

Following incubation, the wells were washed three times with phosphate-buffered saline to remove non-adherent cells and then air-dried. Biofilms were stained with 0.4% (w/v) CV for 15 min, followed by thorough washing. The bound dye was solubilized in absolute ethanol (RCI Labscan, Thailand), and absorbance was measured at 570 nm using a microplate reader (BioTek Instruments, USA). Tetracycline was used as a positive control, and untreated wells (MHB only and bacteria only) served as negative controls. Experiments were performed in triplicate (n = 3).

### Statistical analysis

All experiments were conducted in triplicate, and data are expressed as mean ± standard deviation. Statistical comparisons between treated and control groups were made using one-way analysis of variance followed by Tukey’s *post hoc* test. A p < 0.05 was considered statistically significant. All analyses were conducted using GraphPad Prism version 6.0 (GraphPad Software, La Jolla, CA, USA).

## RESULTS

### GC-MS identification of bioactive compounds

GC-MS analysis identified major compounds such as 5-HMF, 4H-pyran-4-one (2,3-dihydro-3,5-dihydroxy-6-methyl-), loliolide, eugenol, thymol, benzoic acid derivatives, 4-vinylguaiacol, and phytol–all of which are known for their antibiofilm and QS inhibitory (QSI) activities, suggesting their potential in controlling biofilm-associated infections.

GC-MS profiling of *P. foetida* Linn. extracts revealed 30 distinct compounds, based on their retention times, molecular weights, and relative peak areas (Table 1). The most abundant compound was 5-HMF (19.38%), followed by 4H-pyran-4-one, 2,3-dihydro-3,5-dihydroxy-6-methyl- (14.59%). Other notable constituents included loliolide (6.26%), 4-(1-aminoethyl) phenol (4.76%), 2-methoxy-4-vinylphenol (4.53%), and dimethyl sulfone (2.44%).

**Table 1:** Bioactive compounds identified in the ethanolic extract of *Paederia foetida* Linn. identified by GC-MS.

No.	Retention time (min)	Molecular weight (g/mol)	Molecular formula	Compound name	Relative peak area (%)
1	17.1074	126.11	C_6_H_6_O_3_	5-Hydroxymethyl-2-furaldehyde	19.38
2	14.5079	144.12	C_6_H_8_O_4_	4H-Pyran-4-one, 2,3-dihydro-3,5-dihydroxy-6-methyl-	14.59
3	29.5859	196.24	C_11_H_16_O_3_	Loliolide	6.26
4	16.3104	137.18	C_8_H_11_NO	4-(1-Aminoethyl) phenol	4.76
5	18.7548	150.17	C_9_H_10_O_2_	2-Methoxy-4-vinyl-phenol	4.53
6	7.7846	94.13	C_2_H_6_O_2_S	Dimethyl sulfone	2.44
7	14.6095	132.16	C_9_H_8_O	2-Methyl-1-benzofuran	1.87
8	9.2020	144.12	C_6_H_8_O_4_	2,4-Dihydroxy-2,5-dimethyl-3 (2H)-furan-3-one	1.85
9	25.0181	180.20	C_10_H_12_O_3_	4-vinylsyringol	1.50
10	7.8808	98.10	C_5_H_6_O_2_	1,2-Cyclopentanedione	1.31
11	33.3674	256.42	C_16_H_32_O_2_	n-Hexadecanoic acid	1.13
12	53.2646	414.70	C_29_H_50_O	Sitosterol	1.06
13	36.5766	278.40	C_18_H_30_O_2_	9,12,15-Octadecatrienoic acid	0.77
14	15.6151	122.12	C_7_H_6_O_2_	Benzoic acid	0.66
15	52.1574	400.7	C_28_H_48_O	Campesterol	0.63
16	52.5693	412.70	C_29_H_48_O	Stigmasterol	0.60
17	12.1010	128.13	C_6_H_8_O_3_	Furaneol	0.57
18	9.7689	143.23	C_8_H_17_NO	2,2-diethyl-3-methyl-oxazolidine	0.40
19	20.1668	170.25	C_10_H_18_O	8-Hydroxylinalool	0.37
20	19.8833	164.20	C_10_H_12_O_2_	Eugenol	0.31
21	10.8654	108.14	C_7_H_8_O	Benzyl alcohol	0.30
22	24.0821	180.24	C_11_H_16_O_2_	Dihydroactinidiolide	0.29
23	18.8617	138.12	C_7_H_6_O_3_	Salicylic acid	0.28
24	36.4536	280.40	C_18_H_32_O_2_	9,12-Octadecadienoic acid	0.23
25	32.4581	276.40	C_17_H_24_O_3_	7,9-Di-tert-butyl-1-oxaspiro (4,5) deca-6,9-diene-2,8-dione	0.20
26	42.5832	330.50	C_19_H_38_O_4_	2-Palmitoylglycerol	0.19
27	36.9885	284.50	C_18_H_36_O_2_	Octadecanoic acid	0.18
28	51.1465	592.8	C_35_H_60_O_7_	a-Tocopherol	0.17
29	16.5404	154.12	C_7_H_6_O_4_	5-formyl-2-furancarboxylic acid methyl ester	0.12
30	54.1150	426.70	C_30_H_50_O	Cycloartenol	0.10

GC-MS=Gas chromatography-mass spectrometry

Biologically relevant minor constituents included eugenol (0.31%), salicylic acid (0.28%), α-tocopherol (0.17%), and cycloartenol (0.10%). Phytosterols known for their health-promoting properties, such as sitosterol (1.06%), stigmasterol (0.60%), campesterol (0.63%), and cycloartenol, were also detected. Overall, the extract contains a diverse profile of volatile and semi-volatile compounds, including furan derivatives, phenolics, sterols, fatty acids, and other bioactive constituents that may contribute to its therapeutic potential.

### Prediction of 5-HMF, 4H-pyran-4-one derivative (DDMP), and eugenol by ADMET

To assess drug-likeness and safety, *in silico* ADMET predictions were performed for three key bioactive compounds–5-HMF, DDMP, and eugenol–which were identified as major constituents (≥10% abundance) or of known biological relevance. A summary of ADMET and drug-likeness comparisons is provided in Supplementary Table 1.

*In silico* ADMET profiling of 5-HMF indicated favorable pharmacokinetic and drug-like attributes. The compound exhibited high GI absorption, suggesting good oral bioavailability. It was not predicted to be a P-glycoprotein substrate and showed no inhibitory activity toward major cytochrome P450 enzymes (CYP1A2, CYP2C19, CYP2C9, CYP2D6, and CYP3A4), indicating low potential for metabolic interactions or hepatotoxicity.

In terms of water solubility, the ESOL and Ali models classified the compound as very soluble, with log S values of 0.54 and 0.01, respectively. This high solubility enhances its potential for systemic circulation and bioavailability. Drug-likeness evaluation showed that it complies with Lipinski’s rule of five without any violations, indicating good oral drug potential. However, it showed violations in the Ghose and Muegge filters, mainly due to its low molecular weight (126.11 g/mol).

The bioavailability score of the compound was moderate (0.55) and it showed no alerts in Pan-Assay Interference Compounds (PAINS) but had one Brenk alert due to the presence of an aldehyde group, which might raise safety concerns. The compound showed a low log Kp value (7.48 cm/s), indicating minimal skin permeability, and had a synthetic accessibility score of 2.25, implying that it is relatively easy to synthesize. These results support the suitability of DDMP as a drug-like candidate with a low toxicity risk and favorable ADMET properties, making it a promising candidate for further development as an anti-QS or antibiofilm agent.

*In silico* ADMET analysis of DDMP revealed favorable pharmacokinetic and drug-likeness profiles. DDMP exhibited high GI absorption, suggesting favorable oral bioavailability. It was not a P-glycoprotein (P-gp) substrate, indicating a lower likelihood of efflux from cells, and was non-inhibitory to key cytochrome P450 enzymes (CYP1A2, CYP2C19, CYP2C9, CYP2D6, CYP3A4), reducing the risk of metabolic drug–drug interactions or hepatotoxicity.

The compound displayed excellent water solubility across all predictive models (ESOL, Ali, and SILICOS-IT), further supporting its potential for oral administration. It satisfied Lipinski’s rule of five and other drug-likeness filters (Veber, Egan), with only one violation under the Muegge and Ghose filters due to low molecular weight (<160 g/mol).

The bioavailability score was high (0.85), indicating a high probability of systemic availability following oral dosing. In terms of medicinal chemistry, the compound showed zero alerts in both PAINS and Brenk filters and was classified as non-problematic in lead-likeness, with one minor violation due to its low molecular weight. Its synthetic accessibility score was 3.60, indicating moderate ease of synthesis.

The ADMET profile of eugenol (C^10^H^12^O^2^), a phenolic compound widely recognized for its antimicrobial and anti-QS properties, demonstrates promising drug-like characteristics. The compound exhibits high GI absorption, indicating favorable oral bioavailability. In addition, it is not a P-glycoprotein (P-gp) substrate, which suggests that efflux-mediated drug resistance is minimal.

Eugenol was predicted to cross the BBB, suggesting its potential for action in central nervous system-associated infections. Although it does not inhibit other major cytochrome P450 isoenzymes such as CYP2C19, CYP2C9, CYP2D6, or CYP3A4, it shows potential CYP1A2 inhibition, which may require further investigation regarding drug–drug interactions, suggesting a low risk of broad metabolic interference or hepatotoxicity.

Eugenol is classified as soluble across all models (ESOL, Ali, and SILICOS-IT) and exhibits moderate lipophilicity (Consensus Log P_o_w = 2.25), supporting balanced membrane permeability. Its skin permeability (Log Kp) value of −5.69 cm/s also implies moderate topical absorption potential. Eugenol meets the criteria of Lipinski’s Rule of Five without any violations and passes all Veber, Ghose, and Egan filters in terms of drug-likeness.

A minor violation was noted in the Muegge filter due to its low molecular weight (<200 g/mol). A bioavailability score of 0.55 reflects a moderate probability of systemic activity. Eugenol shows no PAINS alerts, indicating a low likelihood of assay interference, although it has one Brenk alert due to the presence of an isolated alkene group.

The compound has a low synthetic accessibility score (1.58), suggesting that it is easy to synthesize, which is beneficial for further development. Eugenol is a drug-like, bioavailable, and low-toxicity phytochemical with strong therapeutic potential as an anti-QS or antibiofilm therapeutic agent.

Overall, 5-HMF exhibits promising pharmacokinetic behavior with acceptable drug-likeness characteristics, making it a suitable candidate for further development of anti-QS drugs.

### Molecular docking analysis of bioactive compounds targeting QS- and biofilm-associated proteins

Molecular docking simulations were conducted to evaluate the binding interactions between bioactive compounds identified from *P. foetida* Linn. extract and key QS and biofilm-associated proteins in *A. baumannii* (AbaI, AbaR, BfmR, PgaA, and PgaB).

Threshold criteria for selecting top-performing docking compounds were based on (i) relative abundance ≥10% in the GC-MS profile and (ii) the lowest binding free energies (ΔG) indicative of strong interaction. For each ligand–protein pair, the docking conformation with the most negative ΔG value was selected because it represents the most thermodynamically favorable pose.

**Figure 1:**
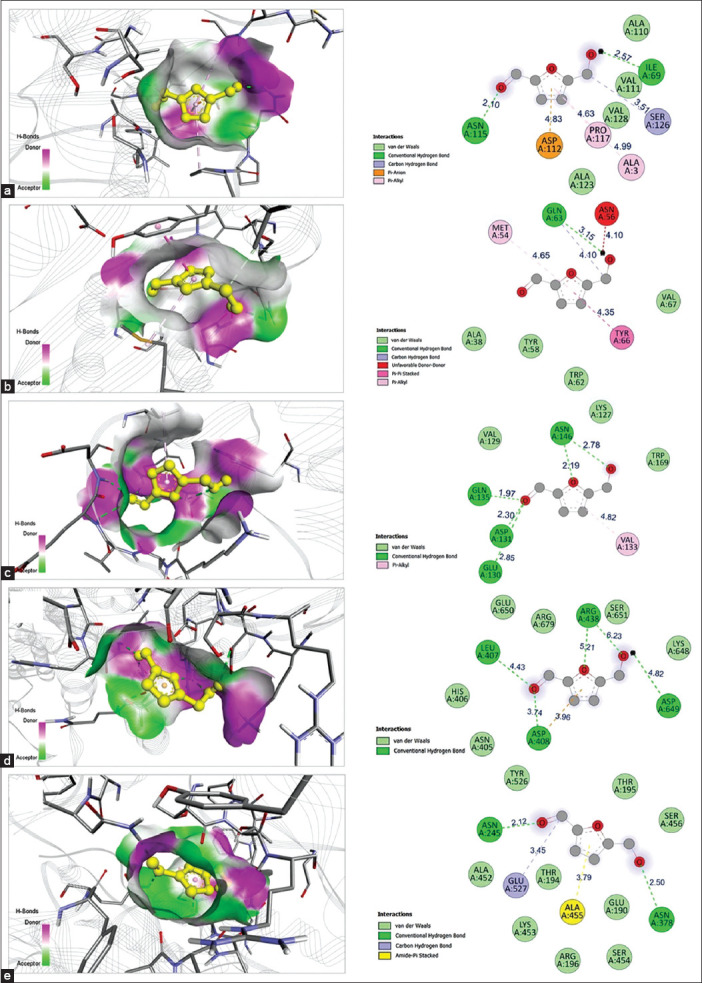
Molecular docking interactions of 5-Hydroxymethyl-2-furaldehyde with five *A. baumannii* biofilm-associated proteins. (a) AbaI, (b) AbaR, (c) BfmR, (d) PgaA, and (e) PgaB. The left panels show the 3D binding conformations of ligand–protein complexes, with ligands displayed in yellow sticks bound within the target protein binding pockets. The corresponding 2D interaction diagrams highlighting hydrogen bonds, Pi-alkyl interactions, and van der Waals forces between ligand and protein residues are shown in the right panels. Interactive bonds are color-coded as follows: green, van der Waals interactions; light green, conventional hydrogen bonds; light pink, carbon hydrogen bonds; magenta, Pi-alkyl interactions; red, electrostatic contacts.

Molecular docking revealed that 5-HMF showed moderate binding affinity against all five targeted proteins, with docking scores of 4.6, 4.8, 4.5, 5.0, and 5.3 kcal/mol (AbaI, AbaR, BfmR, PgaA, and PgaB, respectively). Notably, 5-HMF formed key hydrogen bonds with ILE69 and ASN115 (AbaI), GLN63 (AbaR), GLU130, ASP131, GLN135, and ASN146 (BfmR), and multiple residues in PgaA and PgaB, including ASP649, LEU407, ARG438, ASN245, and ASN378. Additional π-anion, van der Waals, and carbon–hydrogen interactions supported its moderate binding stability within the active sites (Figure 1 and Table 2).

**Table 2:** Molecular docking interactions of AbaI, AbaR, BfmR, PgaA, and PgaB with candidate bioactive compounds targeting biofilm-forming *Acinetobacter baumannii*.

Target proteins	Bioactive compounds	Amino acids involved in the		

		Hydrogen bond	Hydrophobic	Others
AbaI	5-HMF	ILE69, ASN115	ALA3, PRO117, ALA110, VAL111, ALA123, and VAL128	SER126 (C) and ASP112 (E)
	DDMP	ASN115 (2), ALA3 (2)	GLU2, ASP112, ALA123, SER126, PRO127, VAL128, and PRO117	-
	Eugenol	ARG107	LEU36, PRO153, LEU82, PRO153, PHE32, TRP38, LEU105, PHE108, THR150, SER152, VAL156	THR151 (C) and SER106 (C)
AbaR	5-HMF	GLN63	TYR66, MET54, ALA38, TYR58, TRP62, and VAL67	GLN63 (C)
	DDMP	THR77	MET54, TYR66, TYR58, TRP62, ASP75, VAL78, TRP90, PHE103, TRP104, ALA107, LEU112, MET129, and THR131	-
	Eugenol	-	TYR66, TYR58, ALA38, MET54, MET129, ALA38, TYR58, TYR66, GLY40, ASN56, TRP62, GLN63, THR77, VAL78, TRP90, PHE103, THR131,	ASP75 (E)
BfmR	5-HMF	GLU130, ASP131, GLN135, and ASN146 (2)	VAL133, LYS127, VAL129, and TRP169	-
	DDMP	LYS127, ARG149	ARG124, TYR165, ASP166, ARG187, LEU170, and LEU188	TRP169 (C)
	Eugenol	ASN146 (2)	TRP169, LEU170, ARG187, LEU188, LYS127 (2), ARG149, TYR165, and ASP166	-
PgaA	5-HMF	ASP649, LEU407, ASP408, ARG438, and ARG438	A: ASN405, HIS406, LYS648, GLU650, SER651, and ARG679	ASP408 (E)
	DDMP	ARG536 and ARG560 (2)	GLN530, ASN531, ASP535, GLU537, TYR561, GLY562, LEU628, and TYR564	-
	Eugenol	ASN772, GLN760	LYS734, SER732, GLN762, TRP763, GLN764, GLN770, and LEU771	-
PgaB	5-HMF	ASN245, ASN378	GLU527, GLU190, THR194, THR195, ARG196, ALA452, LYS453, SER454, SER456, TYR526, and GLU527	GLU527 (C)
	DDMP	ASN458, PHE529	GLY242, ARG243, ASP264, PHE457, GLU527, PRO528, LEU530, LEU531, and TYR244	-
	Eugenol	ASP265	LEU263, ASP264, ASP265, ASP304, PHE457, ASN458, GLU527, PHE529, and LEU530	-

E represents electrostatic interactions, and C denotes carbon-hydrogen bonds. Numbers in parentheses next to amino acid residues indicate the number of interactions involving that particular residue. DDMP=2,3-dihydro-3,5-dihydroxy-6-methyl-4H-pyran-4-one, 5-HMF=5-Hydroxymethyl-2-furaldehyde, PgaA=AF-A0A2P1B9S4-F1-v4, PgaB=AF-A0A5P9VY74-F1-v4, BfmR=AF-Q2VSW6-F1-v4, AbaI=AF-A0A7S8WE28-F1-v4, AbaR=AF-A0A059ZL64-F1-v4

Compared with 5-HMF, DDMP showed consistently stronger binding affinities, with docking scores of 5.1 kcal/mol (AbaI), 5.4 kcal/mol (AbaR), 5.3 kcal/mol (BfmR), 5.2 kcal/mol (PgaA), and 5.5 kcal/mol (PgaB). It formed multiple hydrogen bonds with ASN115 and ALA3 (AbaI), THR77 (AbaR), LYS127 and ARG149 (BfmR), ARG536 and ARG650 (PgaA), and ASN458 and PHE529 (PgaB). DDMP also exhibited π–π stacked and π–alkyl interactions, indicating strong anchoring within the protein binding pockets.

Eugenol demonstrated the strongest and most consistent binding affinity across all tested QS and biofilm-associated proteins, with docking scores of 5.8 (AbaI), 6.3 (AbaR), 5.2 (BfmR), 5.2 (PgaA), and 5.6 (PgaB) kcal/mol. It interacted through hydrogen bonds (e.g., ARG107 in AbaI, ASN146 in BfmR, and ASP265 in PgaB), as well as extensive hydrophobic and electrostatic contacts involving key residues such as TYR66, MET54, TRP62 (AbaR), TRP169, ARG187 (BfmR), and GLU527, LEU530 (PgaB). These interactions highlight the potential of eugenol as a multi-target inhibitor of QS and biofilm formation in *A. baumannii* (Table 2).

### Antibacterial activity against biofilm-forming *A. baumannii*

The crude extract exhibited antibacterial activity against the biofilm-forming *A. baumannii* strain, with a MIC of 7.81 mg/mL and a MBC of 31.25 mg/mL. These results demonstrate the extract’s potential to inhibit bacterial growth and exert bactericidal effects at higher concentrations.

### Effects of *P. foetida* Linn. extract on *A. baumannii* cell morphology

Field emission SEM (FE-SEM) analysis revealed significant morphological changes in *A. baumannii* following treatment with the crude extract of *P. foetida* Linn. at MIC (7.81 mg/mL) and MBC (31.25 mg/mL) concentrations.

**Figure 2:**
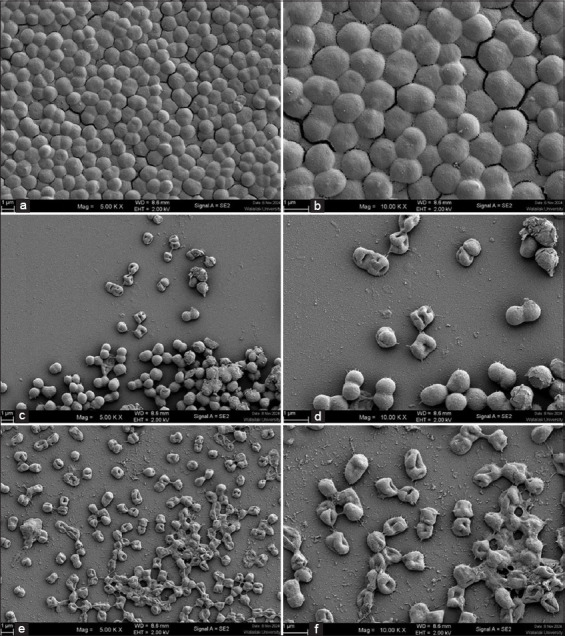
Field emission scanning electron microscopy images of *Acinetobacter baumannii* biofilms treated with *Paederia foetida* Linn. crude extract after 24 h incubation. Panels (a and b) represent untreated controls; (c and d) cells treated with minimum inhibitory concentration (MIC) (7.81 mg/mL); and (e and f) cells treated with minimum bactericidal concentration (MBC) (31.25 mg/mL) of the extract. Untreated cells exhibited intact coccobacillary morphology with smooth surfaces embedded in a dense biofilm matrix. MIC treatment induced morphological alterations, including membrane shrinkage and partial matrix degradation. At MBC, cells displayed severe membrane disruption, surface invaginations, and lysis, with substantial biofilm architecture disintegration. The left panels (a, c, e) were captured at 5,000× magnification, and the right panels (b, d, f) at 10,000× magnification. Scale bars: 1μm.

In the untreated controls (Figure 2a and b), bacterial cells exhibited smooth, intact surfaces characteristic of healthy *A. baumannii* morphology. In contrast, cells treated with the MIC concentration of the extract (Figure 2c and d) displayed visible surface irregularities, partial collapse of cell structure, and membrane disruption, including invaginations and surface roughness.

More extensive damage was observed in cells treated at MBC levels (Figure 2e and f), where bacterial cells exhibited severe membrane deformation, shrin-kage, rupture, and higher incidence of pore formation and cellular debris. These observations indicate that *P. foetida* extract induces dose-dependent ultrastr-uctural damage to the bacterial membrane, disrupting both biofilm integrity and bacterial viability.

### Biofilm formation inhibition capacity

The crude extract demonstrated significant antibiofilm activity against biofilm-forming *A. baumannii* at sub-MIC concentrations. Treatment with ½×, ¼×, and ⅛× MIC concentrations resulted in a dose-dependent reduction in biofilm biomass, as determined by OD at 590 nm measurements.

**Figure 3:**
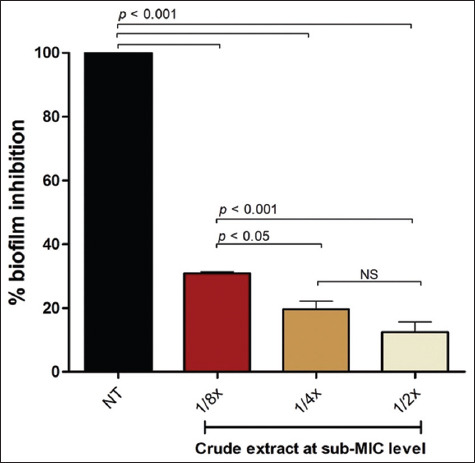
*Paederia foetida* Linn. Extract inhibited *Acinetobacter baumannii* biofilm formation at sub-minimum inhibitory concentration (MIC) levels (½×, ¼×, and ⅛× MIC), as assessed using a static microtiter plate assay. The data represent the average of triplicate experiments ± standard deviation (SD) (n = 3), with error bars indicating SDs. Statistical significance was determined using one-way analysis of variance; *p* values are indicated. Non-significant results are denoted as “NS”. NT= non-treated condition.

The most pronounced inhibition was observed at ½ × MIC, which resulted in a statistically significant reduction in biofilm formation (p < 0.001) compared to the untreated control (Figure 3). While biofilm inhibition was also evident at ¼× and ⅛× MIC, the efficacy was notably reduced at these lower concentrations, indicating diminished effectiveness with decreasing dose.

## DISCUSSION

### One Health Threat of *A. baumannii*

*A. baumannii* has emerged as a critical One Health threat due to its ability to form robust biofilms, exhibit multidrug resistance (MDR), and persist across human, veterinary, and environmental settings. Although traditionally linked to nosocomial infections in humans, its increasing detection in companion animals, veterinary hospital surfaces, and clinical environments highlights its zoonotic potential and risk of environmental dissemination. Genomic similarities between human and animal isolates further support the likelihood of cross-species transmission. Given its classification as a WHO priority pathogen, managing *A. baumannii* necessitates a multidisciplinary appr-oach that incorporates surveillance, antimicrobial stewardship, and infection control measures within the human–animal–environment interface.

### Study objective and methodological approach

To address this One Health concern, the present study investigated the antibacterial, antibiofilm, and QS inhibitory potential of *P. foetida* Linn. extract against *A. baumannii*. A combined approach involving GC-MS-based chemical profiling, *in vitro* phenotypic bioassays, and *in silico* molecular docking and ADMET predictions was employed. The results demonstrate the extract’s multifaceted bioactivity, supporting its potential as a phytotherapeutic agent against drug-resistant *A. baumannii* infections.

### Phytochemical profile and functional insights from GC-MS

GC-MS analysis of *P. foetida* Linn. extract revealed a complex chemical composition, including aldehydes, phenolics, furans, lactones, sterols, fatty acids, and terpenoids. Major constituents such as 5-HMF, DDMP, eugenol, and benzoic acid derivatives are well-documented for their antimicrobial, QS-inhibitory, and antibiofilm properties [8, 28–31]. These compounds hinder bacterial adhesion, metabolic functions, and intercellular communication. Mechanistically, 5-HMF impairs membrane integrity in gram-negative bacteria [32–34], while eugenol disrupts membrane potential, induces intracellular leakage, and binds to QS receptors like AbaR (binding energy: −6.3 kcal/mol) [11, 35–39]. Phenolic compounds, such as 2-methoxy-4-vinylphenol and 4-vinylsyringol, likely act through oxidative stress and lipid peroxidation. In addition, sterols such as stigmasterol, sitosterol, and campesterol may destabilize bacterial membranes [10, 40].

### *In silico* ADMET and drug-likeness analysis

ADMET predictions for 5-HMF, DDMP, and eugenol indicated favorable pharmacokinetics and safety profiles. All three compounds showed high GI absorption and were not P-glycoprotein substrates, reducing the likelihood of efflux-mediated resistance. None showed major CYP450 inhibition, except mild CYP1A2 inhibition by eugenol. DDMP and 5-HMF were highly water-soluble, while eugenol had balanced lipophilicity (Log P = 2.25) and moderate BBB permeability. Drug-likeness scores complied with Lipinski’s Rule of Five, and oral bioavailability ranged from 0.55 to 0.85. Although minor Brenk alerts were noted, these compounds remain viable for further development. These properties enhance their potential as anti-QS or antibiofilm drug candidates [41].

### Molecular docking reveals strong affinity for QS targets

Docking studies showed strong binding affinities between the selected bioactives and QS/biofilm-related proteins (AbaI, AbaR, BfmR, PgaA, and PgaB). Eugenol displayed the highest affinity, particularly with AbaR (−6.3 kcal/mol), implicating its potential as a leading QS inhibitor. These interactions likely inter-fere with AHL synthesis, receptor binding, and signal transduction. Minor compounds such as benzoic acid and furaneol may provide synergistic enhancement of QSI [42, 43].

### Biofilm inhibition and mechanistic implications

Biofilm inhibition assays confirmed significant, dose-dependent reduction in *A. baumannii* biofilm biomass following treatment with the extract. Key compounds, such as DDMP and 5-HMF, likely disrupt early adhesion and EPS production, while their low molecular weights facilitate matrix penetration. This disrupts QS networks, impairs metabolic activity, and reduces EPS production, weakening the biofilm structure. The dual role in bacterial growth suppression and biofilm inhibition underlines the extract’s therapeutic potential [44–47].

### SEM analysis confirms ultrastructural damage

SEM revealed severe ultrastructural changes in *A. baumannii* cells treated with the extract at MIC and MBC concentrations. Untreated cells appeared smooth and intact, while treated cells exhibited membrane rupture, deformation, and signs of structural collapse. This aligns with the extract’s ability to disrupt cell membranes and inhibit peptidoglycan synthesis–mechanisms consistent with known actions of membrane-targeting phytochemicals [17, 44, 48, 49].

### Zoonotic relevance and One Health implications

The detection of *A. baumannii* in dogs, cats, horses, and livestock, along with its persistence in veterinary hospital environments, reinforces its zoonotic potential. Genomic studies support interspecies transmission. Reports from Greece and South Korea further confirm the rising prevalence of *A. baumannii* in veterinary infections [3, 50–52]. These findings underscore the importance of integrated surveillance and infection control across both human and animal health sectors.

## CONCLUSION

This study systematically evaluated the antibacterial, antibiofilm, and anti-QS potential of *P. foetida* Linn. extract against *A. baumannii*, a critical One Health pathogen. GC-MS profiling identified 30 bioactive compounds, including 5-HMF, DDMP, and eugenol, all of which are known for their antimicrobial and QS-inhibitory properties. *In silico* ADMET analysis confirmed favorable drug-likeness profiles for these compounds, characterized by high GI absorption, minimal cytochrome P450 inhibition, and low toxicity risk. Molecular docking studies revealed strong binding affinities of the major compounds to key QS and biofilm-associated proteins (AbaI, AbaR, BfmR, PgaA, and PgaB), with eugenol demonstrating the highest affinity, particularly with AbaR (−6.3 kcal/mol). Phenotypic assays showed significant inhibition of biofilm formation in a dose-dependent manner and confirmed morphological disruptions in bacterial cell structures through SEM. The minimum inhibitory and bactericidal concentrations were determined to be 7.81 mg/mL and 31.25 mg/mL, respectively.

The results highlight the practical potential of *P. foetida* extract as a natural anti-infective agent against biofilm-forming, multidrug-resistant *A. baumannii*. The extract’s dual mechanism of action–targeting both planktonic and biofilm states–offers a novel and sustainable therapeutic approach for veterinary and medical applications. Importantly, its utility aligns with the principles of the One Health framework by addressing the interconnected threats to human, animal, and environmental health posed by antimicrobial resistance.

The study’s strengths include a comprehensive, multi-layered approach combining chemical profiling, phenotypic assays, and computational modeling, as well as the identification and evaluation of both major and minor constituents. However, there are limitations that need to be addressed. *In vivo* efficacy and safety were not assessed, and cytotoxicity evaluations on mammalian cells were not conducted. In addition, the synergistic or antagonistic effects of multiple compounds within the extract remain uncharacterized, and the formulation and stability of the extract require further investigation.

Future research should validate these findings through *in vivo* models, conduct cytotoxicity testing, investigate the combinatorial effects of bioactive constituents, and optimize delivery using nanofor-mulation or encapsulation technologies. Evaluating activity against other ESKAPE pathogens would further strengthen its therapeutic profile.

In summary, *P. foetida* Linn. extract exhibits a broad spectrum of anti-infective activities against MDR *A. baumannii*, driven by multiple bioactive phytochemicals that interfere with QS and biofilm formation. These findings provide a foundation for the development of alternative therapies and contribute to cross-sectoral strategies aimed at combating antimicrobial resistance within the One Health paradigm.

## DATA AVAILABILITY

The supplementary data can be made available from the corresponding author upon request.

## AUTHORS’ CONTRIBUTIONS

SS, TT, and NI: Conceived and designed the study. SS, DH, TK, and TT: Performed the experiments. SS, TT, TN, UP, CK, TN, and NI: Analyzed the data. NI and SS: Statistical analysis. SS and NI: Wrote and edited the manuscript. All authors have read and approved the final version of the manuscript.
